# FACILITATING GROWTH VIA INDEPENDENT LEARNING PLANS FOR PSYCHOLOGISTS TREATING OLDER VETERANS WITH CHRONIC ILLNESS

**DOI:** 10.1093/geroni/igad104.1453

**Published:** 2023-12-21

**Authors:** Jeffrey Gregg, Rachel Rodriguez, Priyanka Mehta, Josea Kramer, Christine Gould

**Affiliations:** Durham VA Medical Center, Durham, North Carolina, United States; Durham VA Health Care System, Durham, North Carolina, United States; VA Palo Alto Health Care System, Palo Alo, California, United States; VA Greater Los Angeles GRECC, Los Angeles, California, United States; VA Palo Alto Health Care System, Palo Alto, California, United States

## Abstract

The VA Geriatric Scholars Program-Psychology Track (GSP) is a national post-licensure geropsychology training program for VA psychologists caring for rural-dwelling older veterans with complex physical, cognitive, and psychological comorbidities. In 2019, GSP developed an advanced workshop model to invest in the continued growth its alumni. The workshop focused on enhancement of geropsychology competencies in treating older veterans with chronic mental and physical illnesses (e.g., PTSD, dementia, diabetes). To facilitate ongoing and individually tailored learning following the workshop, participants developed and executed Independent Learning Plans (ILPs) during a 6-month window with consultation from an experienced geropsychologist. Upon completion, participants submitted final reports and responded to three prompts assessing overall experience, usefulness, and future plans. We examined these qualitative data using a rapid analysis approach with summary templates. Fourteen of 19 participants (73.7%) submitted both final reports and responses to the prompts. All 14 respondents endorsed clinical and/or personal usefulness of the ILPs, with emerging themes including increased confidence, enhanced skills for use with patients with complex needs, and career advancement. Regarding overall experience, most participants noted positive outcomes, including feelings of fulfillment, and noting that ILPs were feasible and learning resources were accessible. A minority (35.7%) identified meaningful barriers most often associated with not having enough time. Future directions included plans to learn new assessment/treatment approaches, read relevant literature, and pursue additional career advancement (e.g., board certification in geropsychology). Overall, our findings suggest ILPs are a viable way to enhance psychologists’ experience and effectiveness in treating older veterans with complex needs.

